# Microstructure and Mechanical Properties of Powder Metallurgy Superalloy Joints Welded by Inertia Friction Welding

**DOI:** 10.3390/ma17061342

**Published:** 2024-03-14

**Authors:** Yongqiang Zhang, Peng Zhao, Yiqi Tong, Honggang Dong, Jun Zhou, Feng Qin, Yanping Bi, Peng Li

**Affiliations:** 1 Harbin Welding Institute Limited Company, Harbin 150028, China; 2 Heilongjiang Advanced Friction Welding Technology and Equipment Key Laboratory, Harbin 150028, China; 3 AECC Commercial Aircraft Engine Co., Ltd., Shanghai 200241, China; 4 School of Materials Science and Engineering, Dalian University of Technology, Dalian 116024, China; 5 Harbin Well Welding Co., Ltd., Harbin 150060, China

**Keywords:** FGH96, inertia friction welding (IFW), microstructure, γ′ phase, microhardness, tensile properties

## Abstract

In recent years, for the structural characteristics and design requirements of the integral rotor and disc shaft of the integrated engine, the welding quality and mechanical properties of superalloy weldments have received increasing attention. In this paper, inertia friction welding (IFW) of FGH96 alloy was carried out using different welding parameters, and the homogeneous connection of FGH96 alloy hollow bars was successfully realized. The microstructure evolution, mechanical properties and fracture failure of the welded joints at room and high temperatures were investigated. The FGH96 alloy IFW joints were divided into the weld nugget zone (WNZ), the thermo-mechanically affected zone (TMAZ), the heat-affected zone (HAZ) and the base metal (BM), and there were significant differences in grain structure and distribution of the γ′ phase in each of the characteristic zones. The microhardness and tensile properties of the IFW joints were investigated, and the results showed an “M”-shaped curve in the microhardness distribution, with the lowest point of hardness observed in the HAZ. The tensile test results indicated that the fracture position moved from the BM to the WNZ with the increase in temperature, the microstructure at the fracture changed significantly and the tensile strength decreased from 1512.0 MPa at room temperature to 1201.3 MPa at 750 °C. The difference in the mechanical properties of the joints was mainly attributed to the changes in the dissolution and precipitation of the γ′ phase.

## 1. Introduction

FGH96 is a second-generation damage-tolerant powder metallurgy superalloy [1]. Compared with the first-generation powder metallurgy superalloy, the content of Ti, Cr, Co, W and Mo is increased, while the content of Al, Nb and C is decreased, so as to reduce the content of γ′ phase and carbides, as well as to adjust the grain size and appropriately reduce the levels of strength. Increasing the content of W and Co can effectively enhance the thermal strength of the alloy. Similarly, elevating the amount of Cr content can improve the oxidation resistance of the alloy. Furthermore, the inclusion of Ce purifies the grain boundaries and improves the toughening effect of the grain boundaries. Therefore, it means FGH96 alloy has excellent comprehensive mechanical properties, as well as excellent corrosion resistance and high-temperature oxidation resistance. It is one of the current superalloys for turbine discs with the highest strength level under operating conditions of 700 °C, and it is the key material for high-performance engine turbine disc rings and other hot components [2,3,4,5]. Inertia friction welding (IFW) utilizes friction heat generated by the relative motion between the contact surfaces of materials, causing the surfaces of the components intended to be welded and the surrounding material to soften and become rapidly forged. This process produces continuous plastic deformation, atomic diffusion and dynamic recrystallization, ultimately achieving dense forging organization. In contrast, the weld in the ordinary fusion welding process is based on cast state organization [6,7,8,9]. The IFW joints do not melt, which fundamentally avoids welding defects linked to melting–solidification and reduces material damage caused by high welding temperatures. In addition, IFW has the advantages of a short welding cycle, fewer welding parameters and higher joint quality, which improves production efficiency and is particularly suitable for the welding of powder superalloys with a high content of γ′ strengthening phases [10,11,12,13,14]. FGH96 alloy inertia friction weldments operate in demanding service environments with complex temperature and stress conditions, which place extremely high requirements on the welding quality and mechanical properties of FGH96 IFW joints. Therefore, the study of the formation process and microstructure evolution of nickel-based superalloy IFW deformed joints has important engineering value and theoretical significance for optimizing the process parameters, improving the quality of the joints and revealing the joint formation mechanism. From a practical perspective, the study provides valuable guidance for connecting rotors and disc shafts in aerospace engines, which can be applied to various connection types, including disc–disc, disc–axis, and integral lobe–disc connections.

Previous studies on IFW have achieved good welded joints of homogeneous or heterogeneous materials involving steel, aluminum alloy, titanium alloy and high-temperature alloy, etc. The studies focused on the physical field, axial shortening, microstructure and mechanical properties of the joints [15,16,17,18,19,20,21,22]. Meanwhile, scholars have explored the effects of welding parameters such as pressure and rotational speed on the microstructure and mechanical properties of the joints [2,23,24]. However, the related research mainly studies the effect of a single parameter on the joint, and the effects of “strong norms” with a large moment of inertia and low rotating speed and “weak norms” with a small moment of inertia and high rotating speed on the joint are rarely analyzed. Therefore, it is of great significance to analyze the effects of “strong norms” and “weak norms” on FGH96 alloy IFW joints. In this paper, for the welded structure requirements of civil aviation engine rotor components, a multi-scale analysis study of FGH96 IFW joints with the same material combination was carried out. The welding heat input was regulated by adjusting the welding process parameters, and the IFW joints were analyzed on multiple scales. The mechanical properties of the joints were tested at room temperature and high temperature. Macroscopic analyses were carried out to determine the bonding condition of the joints and the morphology of the flash. Microscopic analyses were performed to obtain the microstructure changes, grain size, distribution of strengthening phases in the characteristic zones of the joints and the fracture morphology of the test specimens for the mechanical properties after the thermal process of the weld. The multi-scale analysis provides the basis for utilizing homogeneous IFW joints of FGH96 alloy in engineering applications.

## 2. Materials and Methods

The raw materials for the experiment were Ni-based γ′ precipitation strengthening powder metallurgy superalloys FGH96. The nominal chemical composition (wt.%) is shown in Table 1, and the room-temperature tensile properties are shown in Table 2.

**Figure 1:**
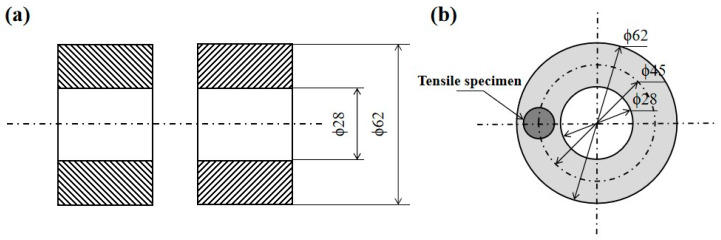
Schematic diagram of workpiece. (**a**) Workpiece dimensions, (**b**) Tensile specimen location (unit: mm).

The weldment was processed into a pipe with an outer diameter of ϕ62 mm and an inner diameter of ϕ28 mm. Its dimensional specifications are shown in Figure 1a. As IFW has the characteristic of different friction heat generation along the radial direction, the distribution of the microstructure and mechanical properties of the welded joints along the radial direction is not uniform. In this test, tensile specimens were obtained at the radial centerline of the weldment, which were used to optimize the welding process parameters and assess the tensile properties of the overall joint, as shown in Figure 1b.

The HWI-IFW-130 axial/radial inertia friction welder was used for the welding experiments. Based on previous research experience, four groups of welding process parameters were selected by varying the initial rotating speed, moment of inertia and welding force, as shown in Table 3, to compare the effects of “strong/weak norms” and pressure on the microstructure and properties of the joints, and to obtain the optimal process parameters.

After the joint specimens were ground and polished, they were chemical etched in a solution of CuCl_2_ (50 g) + HCl (250 mL) + C_2_H_5_OH (250 mL), and the microstructure was observed and analyzed by optical microscope (OM). The strengthening phase morphology of the joints and their distribution pattern were observed and analyzed by scanning electron microscope (SEM) after electrolytic etching with a mixture of H_3_PO_4_ (170 mL) + H_2_SO_4_ (10 mL) + CrO_3_ (15 g). The grain characteristics, crystal orientation and dislocation density of the welded joints were further observed by electron backscatter diffraction (EBSD) after sample preparation by vibratory polishing.

**Figure 2:**
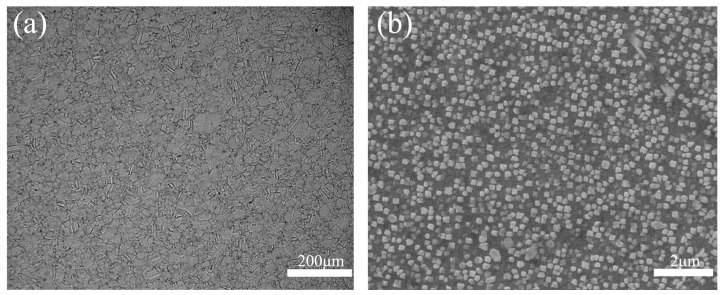
Metallographic microstructure of FGH96 alloy. (**a**) Optical microstructure, (**b**) SEM γ′ phase distribution.

FGH96 alloy is face-centered cubic austenite with low stacking fault. In face-centered cubic metal, the {111} surface is a close-packed twin interface, and the twin interfaces are prone to forming twin crystals by slipping along these close-packed surfaces. The FGH96 alloy matrix is γ phase, and the main strengthening phase is the γ′ phase [25]. The metallurgical microstructure of the base metal (BM) is shown in Figure 2. As seen in the figure, the size of γ′ phase is 80~350 nm.

**Figure 3:**
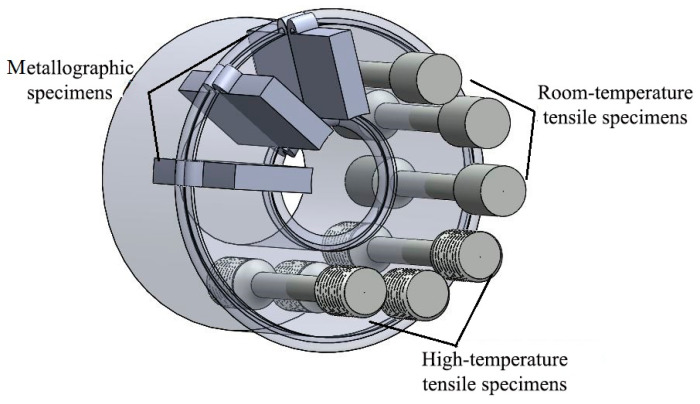
Schematic diagrams of extraction locations and shapes of different analyzed specimens.

**Figure 4:**
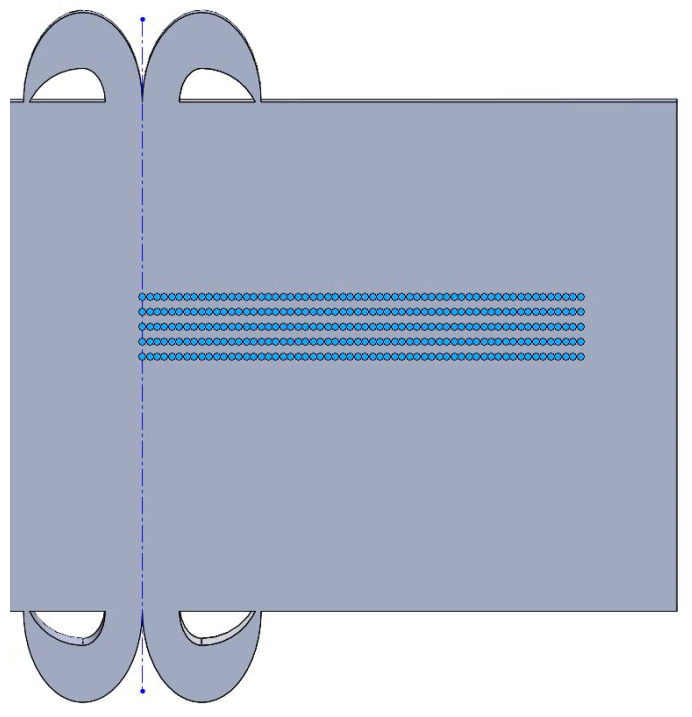
Schematic diagram of microhardness test of FGH96 alloy IFW joints.

Tensile experiments at room temperature and 750 °C were carried out on the mechanical properties of the tensile specimens using a universal testing machine. Three tensile specimens were taken for each parameter. The extraction positions and shapes of the different analyzed specimens are shown in Figure 3. The microhardness test parameters are shown in Table 4. Unilateral hardness was detected at the center of the welded joint along five straight lines parallel to the direction of the joint axis (Figure 4). The average of the five points was taken at the same location, and the measured data were recorded.

## 3. Results and Discussion

In this section, the obtained welded joints were analyzed in terms of macroscopic characteristics, microstructure and γ′ phase distribution, and the microhardness, room-temperature and 750 °C high-temperature tensile properties and fracture morphology of the joints were investigated, so as to establish the link between the microstructure and comprehensive mechanical properties of the joints.

### 3.1. Macrostructure Characteristics of Joints

**Figure 5:**
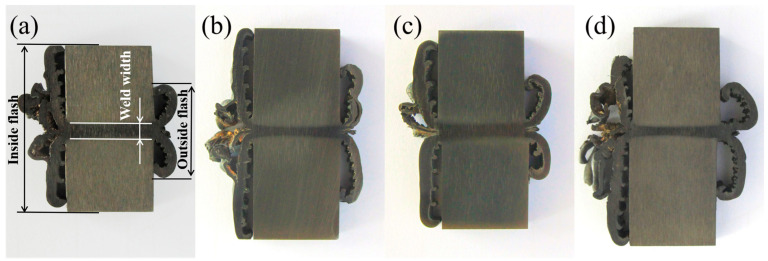
Macrostructure characteristics of FGH96 alloy IFW joints. (**a**) #1, (**b**) #2, (**c**) #3, (**d**) #4.

Figure 5 shows the cross-sectional view of the IFW joints chemically etched with 10% CrO_3_ aqueous solution. Figure 5a–d show the macrostructure characteristics of the welded joints obtained under the four parameters of #1, #2, #3 and #4, respectively. As can be seen from Figure 5, the FGH96 alloy IFW joints had complete flash. The flash size and weld width of each joint are shown in Table 5.

From Figure 5 and Table 5, it can be seen that in the process of IFW, when the moment of inertia and the initial rotating speed remained unchanged, the joint flash increased with an increase in welding force, and the weld width decreased with an increase in welding force. This is due to the fact that as the welding force increases, more metal in the friction interface and its adjacent zone undergoes plastic deformation and the metal flow rate increases, resulting in more metal flowing out of the friction interface and a larger flash. At the same time, more heat will be lost through the flash extrusion. The weld transitioned from a small pressure weld formed along the axial direction by equally thick formations on both bond faces to a double taper weld with a narrow center and wide periphery. For the four groups of parameters in this test, when the welding force remained unchanged, the variations in the weld width of the FGH96 IFW joints obtained by “strong norms” with a large moment of inertia and low rotating speed and by “weak norms” with a small moment of inertia and high rotating speed were very small.

### 3.2. Microstructure Characteristics of Joints

**Figure 6:**
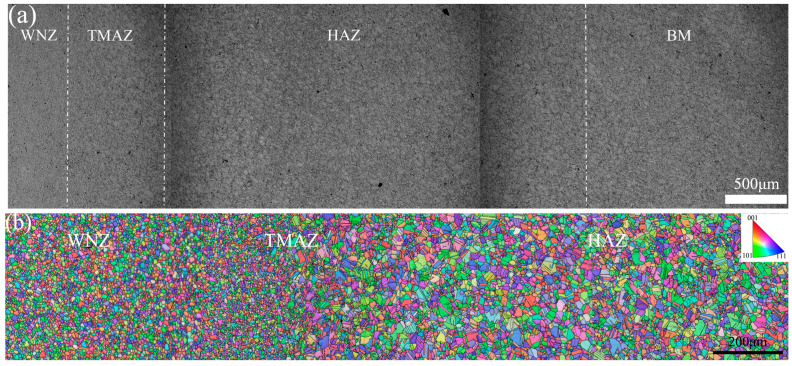
(**a**) OM and (**b**) EBSD microstructure of #4 joint.

It was found that the evolution of the microstructure under the four groups of welding parameters was basically the same, so only the microstructure of #4 joint was selected for analysis. Figure 6a,b show the OM and EBSD microstructure of the FGH96 IFW joint, respectively. The joint was mainly divided into a weld nugget zone (WNZ), thermo-mechanically affected zone (TMAZ), heat-affected zone (HAZ) and BM [17]. The WNZ was in the middle part of the IFW joint, and the grain was fine and uniform equiaxial crystal, with an average grain size of about 6.9 μm, which was a typical dynamic recrystallization grain. The formation of this microstructure is determined by the process characteristics of the IFW deformation process. The friction interface and the surrounding zones during the welding process produce a high shear plastic deformation and rate, leading to the formation of numerous sub-grains, which subsequently act as recrystallization nuclei. Moreover, the maximum temperature in the WNZ during welding surpasses the recrystallization temperature of FGH96 alloy [5], thereby enhancing recrystallization within the WNZ as well as the adjacent structure. By contrast, IFW heating rapidly increases and has a short residence time at a high temperature. This limits grain growth significantly and leads to the transformation of deformed grains in the WNZ into finely recrystallized grains [26]. Coarse and fine crystals coexisted within the TMAZ, with an average grain size of around 7.1 μm, which ranked second to the WNZ in terms of force and heat. A select number of deformed grains recrystallized when subjected to heat, though the degree of grain deformation within this zone was significantly less than that observed in the WNZ. Consequently, the degree and amount of recrystallization within this region were relatively small. The HAZ was a solid solution aging organization that shared a similar grain to that of the BM, but it experienced grain coarsening. The average grain size was approximately 13.5 μm, presenting various degrees of coarsening in each region. This occurs because the HAZ is subjected only to the action of heat and is almost uninfluenced by force. During the cooling process of the weld, the grain grows slowly, resulting in the local area’s grain coarsening.

**Figure 7:**
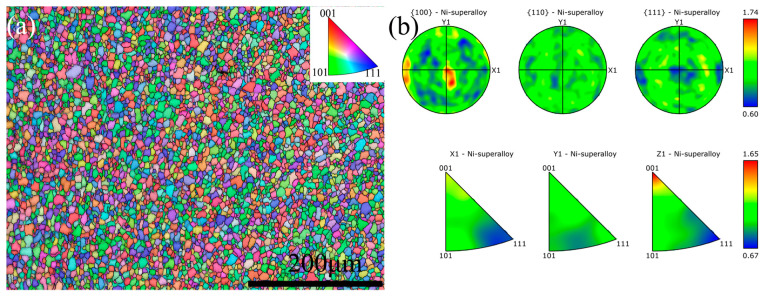
EBSD maps of #4 joint. (**a**) BC + PF map of WNZ, (**b**) PF and IPF maps of WNZ.

Significant deformation existed within the WNZ. However, distinctively deformed grains remained undiscovered. This occurrence is due to the joint’s thermal coupling, allowing for dynamic recrystallization within the WNZ. This process remodels the crystal orientation state of the original BM, ultimately resulting in the distribution of recrystallized grains within the WNZ in the directions of <111>, <101> and <001>, as depicted in Figure 7a. Figure 7b displays the PF and IPF maps of the joint WNZ on the {100}, {110} and {111} crystal planes. The results demonstrated that the maximum crystal texture strength in the WNZ was not more than 1.74 times that of the strength in the complete absence of texture. These findings indicated that the preferred orientation of the crystal texture was weak and the microtexture had a negligible effect on the overall texture strength, which confirmed the promiscuous distribution of the crystals displayed in Figure 7a [4].

**Figure 8:**
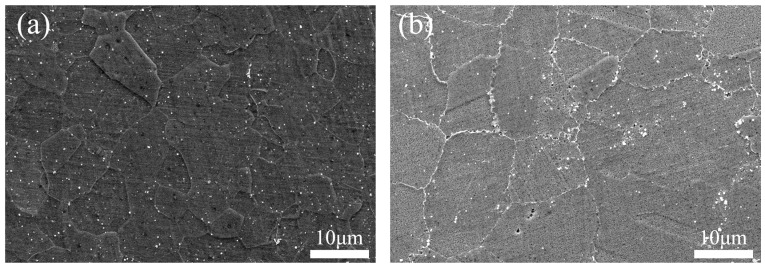
The morphology of grain boundaries in different zones of #4 joint. (**a**) WNZ, (**b**) BM.

After the welding process, the fast cooling rate prevented the large γ′ phase from precipitating at the grain boundaries. This resulted in the grain boundaries in the WNZ becoming relatively straight, as depicted in Figure 8a. Meanwhile, the grain boundaries in the BM took on a “sawtooth” shape, as shown in Figure 8b. These “sawtooth”-shaped grain boundaries can inhibit the relative sliding between the grain boundaries during plastic deformation, leading to enhanced strength, specifically creep strength. Flat grain boundaries could potentially contribute to significant plastic deformation at the weld of the welded specimen.

**Figure 9:**
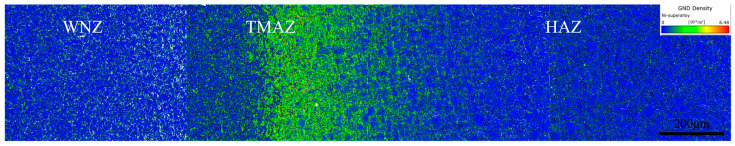
GND density map of #4 joint.

During the process of IFW, a significant amount of deformation energy is produced at the weld, which then gets stored within the metal matrix as dislocations. The dislocation density is directly proportional to the amount of energy generated. Dislocations at grain boundaries will have a greater impact on matrix strengthening. The sub-grain boundaries in the grains are mainly formed by the ordering of edge-type dislocations, and the greater the dislocation density, the larger the orientation difference between the sub-grains. Therefore, the change in sub-grain orientation difference in different zones of the specimen can be measured by EBSD, which indirectly reflects the change in dislocation density. Figure 9 shows the GND density map of the FGH96 IFW joint, demonstrating that the TMAZ had the highest dislocation density with a large crystal orientation difference. Due to the friction torque, significant plastic deformation occurs in the WNZ and TMAZ, leading to the generation of a vast number of dislocations that anchor the grain boundaries. In the WNZ, the dislocation density is reduced because of the complete dynamic recrystallization [26]. Under higher temperatures, the HAZ reduced dislocation density via grain growth, thereby diminishing the dislocation strengthening effect.

### 3.3. Evolution of the γ′ Phase Distribution

**Figure 10:**
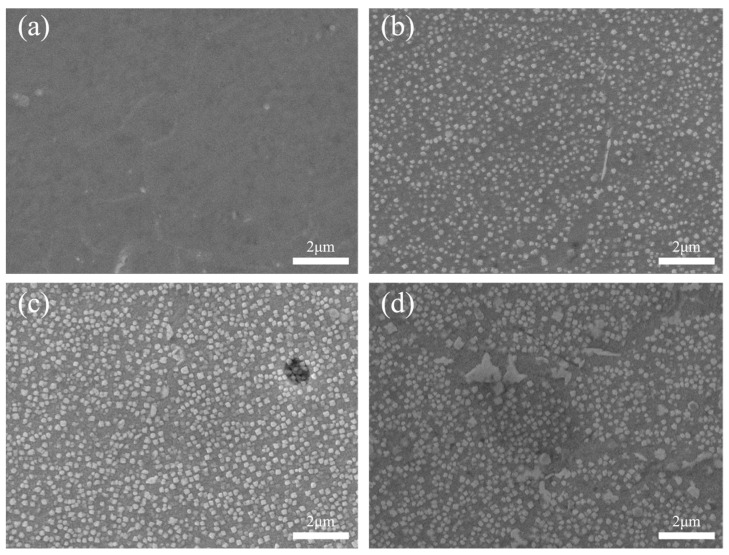
Distribution of γ′ phase in each characteristic zone of #4 joint. (**a**) WNZ, (**b**) TMAZ, (**c**) HAZ, (**d**) BM.

Figure 10 depicts the distribution of the γ′ phase in various zones of the FGH96 IFW joint. The results exhibited significant changes in both the size and shape of the γ’ phase while moving away from the WNZ. There was no presence of a γ′ strengthening phase in the fine-grained microstructures of the WNZ. This occurs because IFW heating is characterized by rapid, instantaneous and localized heating. Once friction ceases, the heat in the joint dissipates quickly through air convection and heat conduction, causing the temperature to promptly decrease to a level below the precipitation temperature of the strengthening phase. During the IFW process, the weld temperature can reach up to 1300 °C, which is considerably higher than the solid solution temperature of the γ′ phase (1110~1120 °C). This leads to the complete dissolution of the γ′ strengthening phase within the matrix. Consequently, the strengthening phase is practically absent in the central region of the joints where the fine-grained microstructures are formed. The γ′ strengthening phase in the TMAZ was partly dissolved within the matrix. Due to the high temperature in the central zone of the joint, this area surpasses the solid solution temperature of γ′, leading to partial dissolution of the γ′ strengthening phase within the TMAZ. This is due to the brief exposure of the area’s temperature above the solid solution temperature of γ′, followed by the temperature instantly dropping below the precipitation temperature of the γ′ phase. The dissolved strengthening phase within the matrix does not precipitate out. As a result, the number of strengthening phases present is less than that of the BM. The γ′ strengthening phase dissolved in small quantities within the HAZ and underwent minimal change. This can be attributed to the temperature having just reached the solid solution temperature of the γ′ strengthening phase. As a result, γ′ can dissolve only in small amounts within the matrix [3].

### 3.4. Tensile Failure Analysis of Joints

Table 6 displays the average values of the room-temperature tensile properties of the joints with different welding parameters, mainly including tensile strength, yield strength, elongation and section shrinkage. Tensile strength and yield strength exhibited little variation across the four welding parameters, stabilizing at approximately 1512.0 MPa and 1169.4 MPa, respectively. Likewise, the elongation and section shrinkage did not differ significantly, remaining at roughly 17.4% and 32.8%, respectively. It was shown that for powder metallurgy superalloys, IFW had excellent process adaptability and a wide process window, and high quality and stable joints can be obtained within a wide range of process parameters. Furthermore, the tensile strength and yield strength of the joints achieved 94.7% and 96.2% of the BM, respectively, with the elongation and section shrinkage reaching 85.3% and 137.8% of the BM. This is due to the fact that IFW joints are formed under the combined action of thrust and torque, causing viscoplastic deformation and recrystallization of the weld metal. As a result, an ultra-fine grain is forged in the friction weld. Since IFW is a solid-state welding process, it does not result in the metallurgical defects associated with melting and solidification. As the IFW joint features overall synchronous and uniform heating, with uniform heat and force, fast heat input and a small heat deformation zone, it results in a high-quality IFW joint with excellent mechanical properties [27].

Table 7 shows the average values of the high-temperature tensile properties of the joints at 750 °C under different welding parameters, which mainly include tensile strength, yield strength, elongation and section shrinkage. No significant differences in high-temperature tensile strength, yield strength, elongation and section shrinkage were observed for the four different welding parameters. Comparison of Table 6 and Table 7 shows that as the temperature increased, the tensile strength decreased from 1512.0 MPa at room temperature to 1201.3 MPa, the yield strength decreased from 1169.4 MPa at room temperature to 1004.1 MPa, the elongation decreased from 17.4% at room temperature to 5.1% and the section shrinkage decreased from 32.8% at room temperature to 7.3%. From the high-temperature tensile results, #3 joint has better high-temperature performance, with a tensile strength of 1223.0 MPa at 750 °C.

**Figure 11:**
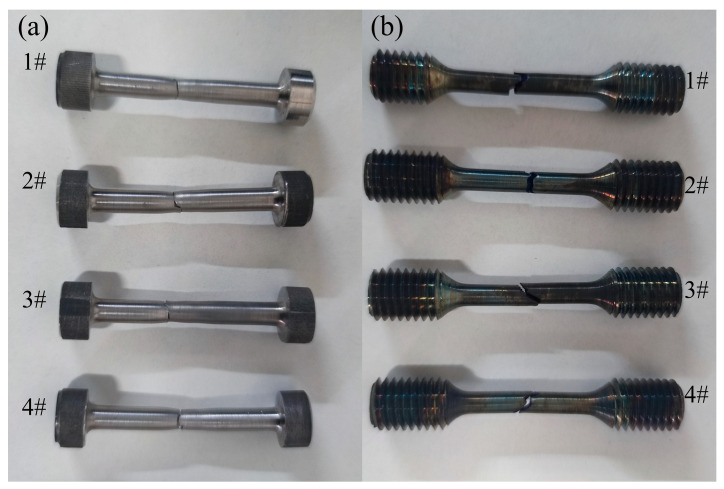
FGH96 alloy IFW joints’ (**a**) room-temperature and (**b**) high-temperature tensile fracture locations.

Figure 11 shows the fracture position of the room-/high-temperature tensile specimens of the FGH96 alloy IFW joints, which were subjected to different welding parameters. Significant necking was observed in the welded specimens with the fracture located in the HAZ. The IFW joint has a WNZ of distorted dynamic recrystallized structure, and a TMAZ with a small amount of dynamic recrystallized structure and a large amount of deformed structure with a large amount of distortion, which are relatively superior in strength. The grain in the HAZ grows at high temperature, while its strengthening phase dissolves in the matrix in small quantities. Consequently, the strength is lower than that of the BM, and the specimen deforms plastically in tension. Owing to the lowest strength in the HAZ, the plastic deformation is mainly concentrated in the HAZ, and a pile-up of dislocation occurs in the region where the coarse/fine crystal interface creates the crack source and fracture occurs [28]. The high-temperature tensile failure of the joint primarily occurred within the WNZ. This is because the high welding temperature reaches the solid solution temperature of the alloy’s γ′ strengthening phase. Consequently, the γ′ phase dissolves in the matrix. The FGH96 alloy mainly relies on the γ′ phase for the precipitation strengthening effect, and when it dissolves in the matrix, the alloy loses its strengthening effect. This results in a rapid decline in strength [5].

**Figure 12:**
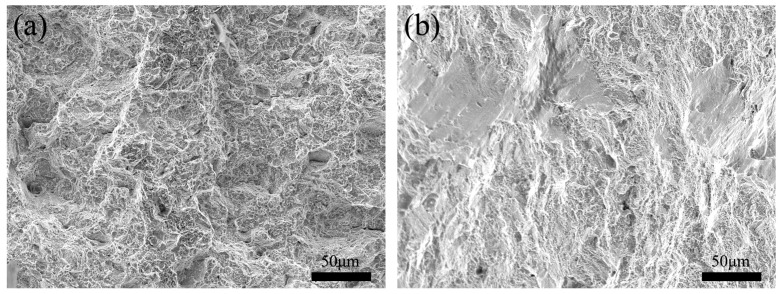
FGH96 alloy IFW #4 joint’s (**a**) room-temperature and (**b**) high-temperature tensile fracture morphology.

Figure 12 shows the room-/high-temperature tensile specimen fractures of #4 joint. The room-temperature tensile fracture of the welded joints can be seen with obvious plastic fracture characteristics, the existence of large dimples, while the existence of a small number of small facets and intergranular cracks can be seen; it can therefore be judged that the fracture mode of the joint was dominated by ductile fracture. The presence of shallow dimples and cleavage planes of different sizes at the high-temperature tensile fracture of the joints suggested that the high-temperature tensile fracture mode of the joints was a mixed fracture [8,29].

### 3.5. Microhardness

**Figure 13:**
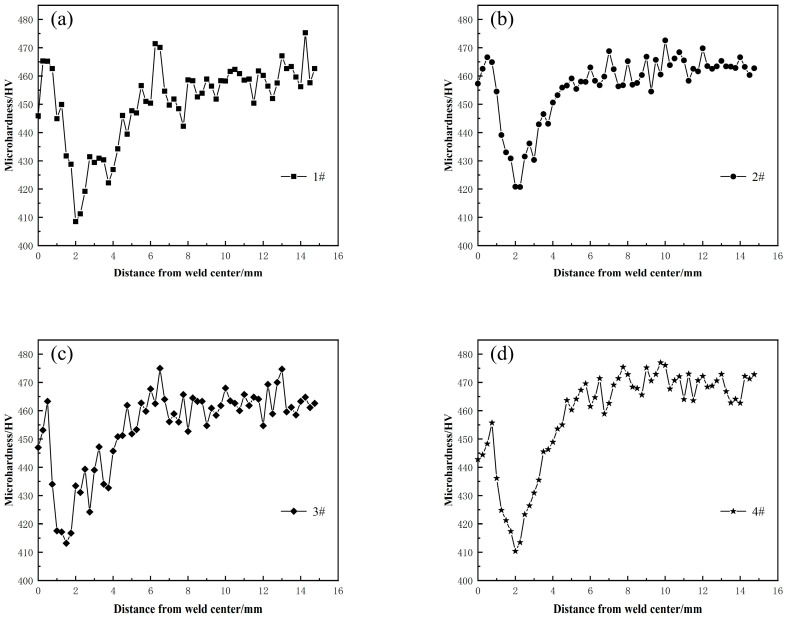
Microhardness of FGH96 alloy IFW joints. (**a**) #1, (**b**) #2, (**c**) #3, (**d**) #4.

Microhardness is influenced by the grain size, number and size of the γ′ strengthening phase, and distortion of the grain. The microhardness curve of the FGH96 IFW joint exemplifies the integrated strengthening effect of these factors. It corresponds directly to changes in strength. Figure 13 shows the microhardness distribution curves of the FGH96 alloy IFW joint under different welding parameters. As depicted in Figure 13, the welded joints exhibited a significant gradient in hardness, with the curves displaying an “M” shape. The microhardness of the WNZ matched or fell below that of the BM. The microhardness of the TMAZ was equal to or greater than that of the BM. The microhardness of the HAZ was lower than that of the BM, and the weakest point of hardness was found within the HAZ.

Figure 13 shows that the region with the highest degree of hardness was approximately 0.5 mm from the joint’s center, which is due to the fact that the region of 0.5 mm from the center of the joint is located in the TMAZ. The microstructure of the TMAZ was mainly dominated by deformation grains, and the γ′ strengthening phase was only partially dissolved in the matrix. The precipitation strengthening and lattice distortion strengthening of the γ′ phase made the microhardness of this place appear in the peak value. The WNZ was situated at the center of the joint. It consisted of finely dynamic recrystallization grains, but the secondary γ′ phase was almost completely dissolved in the matrix. Thus, when the elevating impact of fine grain strengthening and the weakening influence of γ′ precipitation strengthening were combined, the microhardness curve exhibited a trough. The hardness was lowest in the area 2 mm from the joint center, which was the HAZ, where grain coarsening occurred during the welding process, and the degree of grain coarsening varied from region to region, with low grain boundary strengthening and dislocation strengthening levels. As a result, there are varying degrees of hardness reduction [1,30].

## 4. Conclusions

In this paper, the microstructure evolution of each characteristic zone of FGH96 alloy IFW joints under different welding parameters (welding force, initial rotating speed, moment of inertia) was investigated. And mechanical property tests were carried out, including microhardness analysis of the joints, room-temperature and high-temperature tensile mechanical properties, and failure analyses. The results of the study are of reference significance for the application of FGH96 alloy weldments and are favorable for the application of FGH96 alloy in the connection of aero-engine rotors and disc shafts. The main conclusions are as follows.

The FGH96 alloy IFW joints were mainly divided into the WNZ, TMAZ, HAZ and BM. The grains in the WNZ were very fine and uniform equiaxial crystals. The crystals were haphazardly distributed, the strength of the preferred orientation of the crystal texture was negligible and the γ′ strengthening phase was almost completely dissolved. The coexistence of coarse and fine crystals can be observed in the TMAZ, and the γ′ strengthening phase was partially dissolved in the matrix. The dislocation density was found to be highest in the TMAZ. The grains in the HAZ were coarsened, and the γ′ strengthening phase was dissolved in a small amount in the matrix and remained almost unchanged.

The room-temperature tensile properties of the FGH96 alloy IFW joints under four sets of welding parameters were basically the same, with tensile strength and yield strength stabilized at 1512.0 MPa and 1169.4 MPa, respectively, which were 94.7% and 96.2% of those of the BM. There was no significant difference in the high-temperature tensile properties of the joints under the four different welding parameters, and the tensile strength and yield strength were maintained at 1201.3 MPa and 1004.1 MPa, respectively. When the initial rotating speed was 645 r/min, the moment of inertia was 388 kg∙m^2^ and the welding force was 320 MPa, the tensile strength at 750 °C reached a maximum value of 1223.0 MPa.

The FGH96 alloy IFW joints fractured in the HAZ at room temperature, showing typical ductile fracture characteristics. They fractured in the WNZ at 750 °C, and the fracture mode was a mixed fracture.

The microhardness distribution curves of the FGH96 alloy IFW joints under different welding parameters showed an “M” shape. The microhardness of the WNZ was equivalent to or less than that of the BM. The microhardness of the TMAZ was equivalent to or greater than that of the BM. The microhardness of the HAZ was less than that of the BM, presenting a minimum level of hardness.

## Figures and Tables

**Table 1:** Chemical composition of FGH96 alloy (wt.%).

Element	Cr	Co	Mo	W	Al	Ti	Ta	Nb	Zr	Ce	C	B	Ni
Content	16.0	13.1	4.0	3.8	2.4	3.6	0.02	0.7	0.04	0.01	0.04	0.01	Bal.

**Table 2:** Room-temperature tensile properties of FGH96 alloy.

Tensile Strength, MPa	Yield Strength, MPa	Elongation, %	Section Shrinkage, %
1596.0	1216.1	20.4	23.8

**Table 3:** FGH96 alloy IFW welding process parameters.

Serial Number	Initial Rotating Speed, r/min	Moment of Inertia, kg·m^2^	Welding Force, MPa
#1	770	280	320
#2	770	280	405
#3	645	388	320
#4	645	388	405

**Table 4:** Microhardness test parameters used in this study.

Base Metals	Load, g	Pressure Holding Time, s	Vertical Weld Interval, mm	Deployment	Parallel Weld Interval, mm	Number of Steps
FGH96	100	15	0.25	60	0.5	5

**Table 5:** Macrostructure characteristics of FGH96 alloy IFW joints under different welding parameters.

Welding Process Parameters				Macrostructure Characteristics of Joint		
Serial Number	Initial Rotating Speed, r/min	Moment of Inertia, kg∙m^2^	Welding Force, MPa	Outside Flash, mm	Inside Flash, mm	Weld Width, mm
#1	770	280	320	18.5	31.9	2.9
#2	770	280	405	27.5	41.6	2.0
#3	645	388	320	20.9	36.8	2.8
#4	645	388	405	23.4	37.4	2.0

**Table 6:** Room-temperature tensile properties of FGH96 alloy IFW joints under different welding process parameters.

Welding Process Parameters				Room-Temperature Tensile Properties of Joint			
Serial Number	Initial Rotating Speed, r/min	Moment of Inertia, kg∙m^2^	Welding Force, MPa	Tensile Strength, MPa	Yield Strength, MPa	Elongation, %	Section Shrinkage, %
#1	770	280	320	1516.9	1189.5	17.7	37.8
#2	770	280	405	1510.4	1165.6	16.8	33.5
#3	645	388	320	1514.0	1164.7	16.9	28.1
#4	645	388	405	1506.8	1157.6	18.2	31.6

**Table 7:** High-temperature tensile properties of FGH96 alloy IFW joints under different welding process parameters.

Welding Process Parameters				High-Temperature Tensile Properties of Joint			
Serial Number	Initial Rotating Speed, r/min	Moment of Inertia, kg∙m^2^	Welding Force, MPa	Tensile Strength, MPa	Yield Strength, MPa	Elongation, %	Section Shrinkage, %
#1	770	280	320	1180.0	999.0	4.9	7.0
#2	770	280	405	1201.5	993.5	5.7	5.9
#3	645	388	320	1223.0	1006.0	6.8	9.0
#4	645	388	405	1200.5	1018.0	3.1	7.1

## Data Availability

Data are contained within the article.
